# RIPK1 in Alzheimer’s Disease: A Context-Dependent Signaling Hub Linking Aβ, Tau, Neuroinflammation, and Regulated Cell Death

**DOI:** 10.3390/cells15181692

**Published:** 2026-09-18

**Authors:** Antonella Caccamo, Marika Lanza, Giovanna Casili, Salvatore Oddo

**Affiliations:** Department of Chemical, Biological, Pharmaceutical and Environmental Sciences, University of Messina, Viale Ferdinando Stagno D’Alcontres 31, 98166 Messina, Italy; antonella.caccamo@unime.it (A.C.); giovanna.casili@unime.it (G.C.)

**Keywords:** necroptosis, microglia, TNFR1 signaling, RIPK3–MLKL pathway

## Abstract

Alzheimer’s disease is a multifactorial neurodegenerative disorder in which amyloid-β accumulation, tau pathology, neuroinflammation, synaptic dysfunction, vascular injury, and neuronal loss interact across multiple cellular compartments. Although amyloid and tau remain central to disease definition and biomarker staging, growing evidence indicates that inflammatory and regulated cell-death pathways actively shape disease progression. Receptor-interacting protein kinase 1 (RIPK1) has emerged as a context-dependent signaling checkpoint that links inflammatory signaling to cell-fate decisions. In its scaffold and ubiquitinated forms, RIPK1 supports TNFR1-associated NF-κB and MAPK signaling, whereas RIPK1 kinase activation can promote apoptosis and RIPK1-RIPK3-MLKL-dependent necroptosis when regulatory checkpoints fail. In Alzheimer’s disease, RIPK1 signaling has been implicated in disease-associated microglial responses, impaired amyloid-β handling, astrocyte reactivity, tau-associated neuronal stress, neurovascular dysfunction, and necroptotic cell death. Here, we review the molecular regulation of RIPK1; evaluate evidence from human tissue, experimental models, and cellular systems; and discuss how RIPK1 may function as a convergence and amplification node linking amyloid-β, tau, neuroinflammation, and regulated cell death. We also examine the therapeutic rationale for targeting RIPK1, including CNS-penetrant inhibitors, biomarker challenges, timing of intervention, patient selection, and safety considerations. We propose that RIPK1 is an understudied pathway in Alzheimer’s disease pathogenesis and that defining its cell-type-specific and stage-dependent functions will be essential to determine whether RIPK1 inhibition can be developed as a disease-modifying therapeutic strategy.

## 1. Introduction

Alzheimer’s disease (AD) remains the most common cause of dementia and one of the major medical challenges of aging societies. Pathologically, AD is defined by extracellular amyloid-beta (Aβ) plaques and intracellular tau-containing neurofibrillary tangles, while clinically it progresses from subtle cognitive changes to dementia and loss of independence. The NIA-AA research framework has helped move the field toward a biological definition of AD based on Aβ, tau, and neurodegeneration biomarkers. Still, this framework does not by itself explain how these lesions produce chronic glial activation, synaptic failure, and neuronal loss [1,2,3].

The classic Aβ- and tau-centered model has therefore expanded into a broader view of AD as a multicellular disorder. Aβ and tau remain central to diagnosis and pathobiology, but they interact with inflammatory, vascular, metabolic, and cell-death pathways that shape the temporal and regional pattern of neurodegeneration. This shift matters because many downstream mechanisms may determine whether Aβ and tau pathology remains relatively contained or progresses to synaptic dysfunction and neuronal death. Neuroinflammation has become especially important in this revised model: activated microglia and astrocytes can help clear debris and contain injury, but chronic or maladaptive activation can amplify cytokine signaling, complement activation, oxidative stress, and neuronal vulnerability [4,5,6].

Human genetics and single-cell studies strongly support this cellular-ecosystem view of AD. Large genetic studies have implicated pathways related to Aβ and tau biology, but also lipid metabolism, innate immunity, endocytosis, myeloid signaling, and inflammatory receptor pathways [7,8]. Rare coding variants in TREM2, PLCG2, and ABI3 further point to microglial-mediated innate immunity as a key determinant of AD risk [9]. In parallel, single-cell and single-nucleus transcriptomic studies have shown that AD alters multiple brain cell types and that microglia can transition into disease-associated states near plaques and other sites of tissue injury [10,11,12]. Together, these findings indicate that AD cannot be understood solely as protein aggregation followed by passive neuronal loss. Instead, AD involves active signaling programs that coordinate glial reactivity, inflammatory amplification, proteostatic stress, and regulated forms of cell death.

A central question is which signaling nodes connect these processes. The field needs mechanisms that can explain how persistent inflammatory receptor activation, glial state transitions, and neuronal death converge during disease progression. Receptor-interacting protein kinase 1 (RIPK1) is well positioned to fill this gap. RIPK1 functions downstream of tumor necrosis factor receptor 1 (TNFR1) and other inflammatory receptors, where it can support nuclear factor-kB (NF-kB) and mitogen-activated protein kinase signaling through scaffold- and ubiquitin-dependent mechanisms. Under different checkpoint conditions, RIPK1 kinase activity can instead promote RIPK1-dependent apoptosis or drive RIPK1-RIPK3-mixed lineage kinase domain-like protein (MLKL) necroptosis, a lytic form of regulated cell death that can release damage-associated molecular patterns and intensify inflammation [13,14].

RIPK1 is therefore often described as a necroptosis kinase, but that description is too narrow for AD. RIPK1 is a context-dependent signaling checkpoint in AD that controls two partially separable processes: a kinase/scaffold-dependent inflammatory glial program and a kinase-dependent regulated cell death pathway. Understanding when, where, and in which cell type RIPK1 is activated will determine whether RIPK1 inhibition is therapeutically useful. This distinction is particularly relevant in the AD brain, where the same molecule may regulate inflammatory transcription in microglia and astrocytes, death signaling in vulnerable neurons, and possibly vascular or oligodendroglial responses depending on disease stage and local stress signals.

This narrative review is mainly based on two key lines of evidence. First, RIPK1 has been linked to disease-associated microglial responses and lysosomal dysfunction in AD models, suggesting that it can influence Aβ accumulation and glial inflammatory states rather than acting only at the final stage of cell death [15]. Second, we reported necroptosis activation in human AD brain and AD mouse models, with markers of RIPK1-RIPK3-MLKL signaling correlating with neuropathological severity and cognitive decline in AD patients [16]. These findings place RIPK1 at the intersection of two major unresolved problems in AD: how glial inflammation becomes maladaptive and how neurons die during disease progression.

In this narrative review, we develop the idea that RIPK1 signaling is an understudied pathway that may be involved in many aspects of AD pathogenesis. We first summarize the signaling logic of RIPK1, emphasizing the distinction between scaffold-dependent inflammatory signaling and kinase-dependent death signaling. We then examine evidence for RIPK1 activation in human AD, animal models, and cellular systems; discuss cell-type-specific roles in microglia, astrocytes, neurons, oligodendrocytes, and vascular cells; and evaluate the therapeutic rationale and limitations of targeting RIPK1. Our goal is not to present RIPK1 as a single master cause of AD, but to define it as a candidate convergence point whose contribution may depend on disease stage, cellular context, and the balance between protective and pathogenic inflammatory responses.

We identified relevant literature through PubMed searches. We prioritized peer-reviewed studies that examined RIPK1 expression or activation, genetically or pharmacologically manipulated RIPK1; evaluated RIPK3–MLKL signaling or necroptosis in human AD tissue and AD-relevant experimental models; or investigated CNS-penetrant RIPK1 inhibitors in clinical studies. Because we designed this article as a narrative review, we did not use a prespecified systematic-review protocol or perform a formal risk-of-bias assessment.

## 2. RIPK1 Signaling Logic: Scaffold Function, Kinase Activity, and Cell-Fate Checkpoints

**Figure 1:**
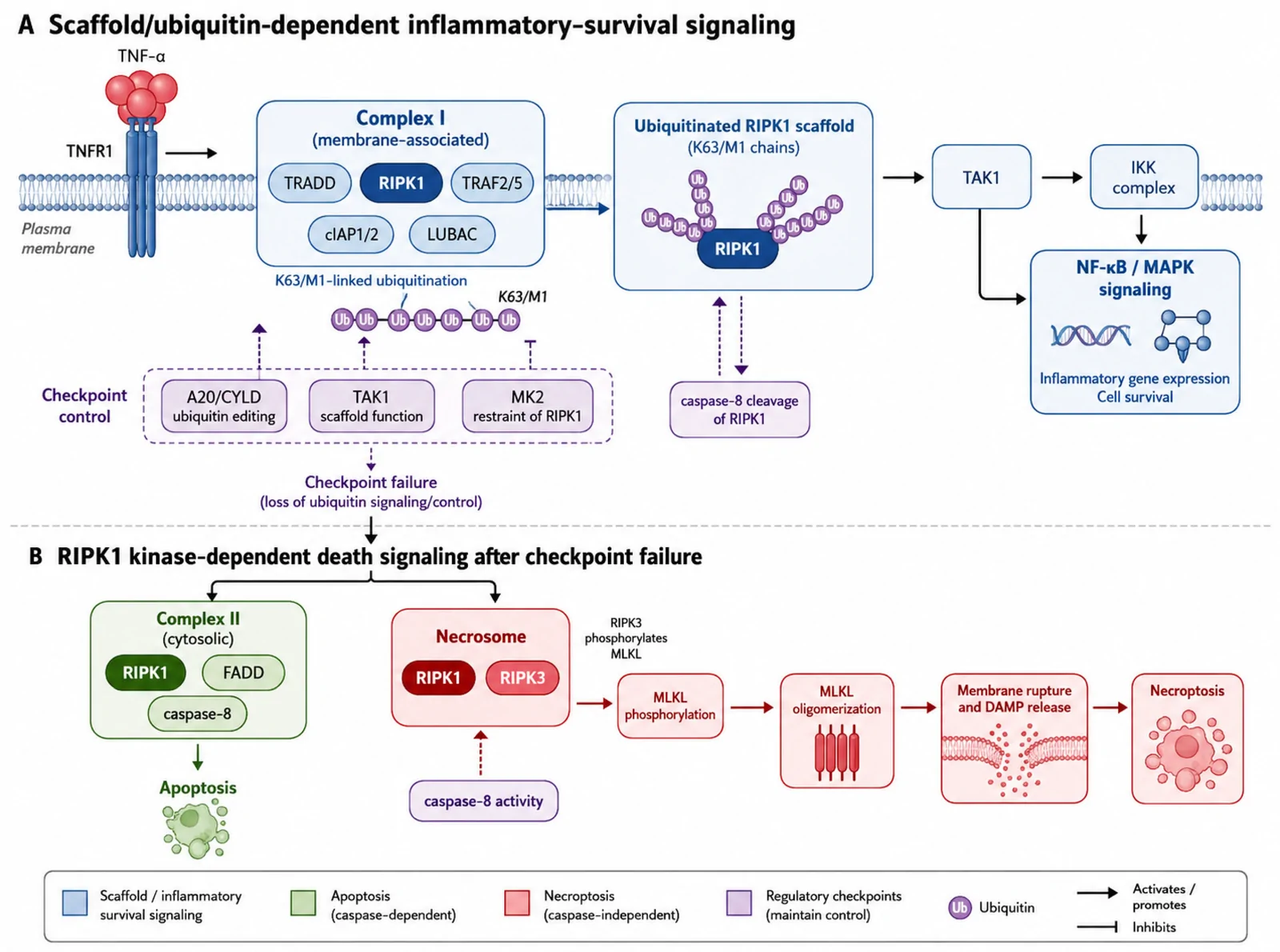
RIPK1 signaling links scaffold-dependent inflammatory signaling to kinase-dependent cell death. TNF-α engagement of TNFR1 promotes the assembly of membrane-associated complex I, in which ubiquitinated RIPK1 acts as a scaffold to support TAK1-, IKK-, NF-κB, and MAPK-dependent inflammatory and survival signaling. Regulatory checkpoints, including A20/CYLD-mediated ubiquitin editing, TAK1, MK2, and caspase-8-mediated RIPK1 cleavage, restrain RIPK1 activation. When these checkpoints fail, RIPK1 shifts toward kinase-dependent cytosolic death signaling through complex II-mediated apoptosis or RIPK1-RIPK3-MLKL-dependent necroptosis, resulting in membrane rupture and DAMP release.

RIPK1 is often introduced as a necroptosis kinase, but that description is too narrow for a review focused on AD. RIPK1 acts as a multidomain signaling checkpoint that can promote inflammatory gene expression, preserve cell survival, trigger caspase-8-dependent apoptosis, or engage RIPK3–MLKL-dependent necroptosis. These outputs depend less on RIPK1 expression alone than on the receptor context, the ubiquitin landscape, phosphorylation state, caspase-8 activity, and the cell type in which the pathway is activated. This distinction is central to AD because RIPK1 may contribute to glial inflammation without necessarily causing cell death, while in stressed neurons or glia the same pathway may become cytotoxic [13,14] (Figure 1).

### 2.1. Domain Organization and Modular Signaling Capacity

Human RIPK1 is organized into three main functional regions: an N-terminal serine/threonine kinase domain, an intermediate region that contains the RIP homotypic interaction motif (RHIM) and several key regulatory residues, and a C-terminal death domain (DD). The kinase domain mediates RIPK1 catalytic activity and is required for RIPK1-dependent apoptosis and for many forms of RIPK1-dependent necroptosis. The RHIM enables homotypic interactions with other RHIM-containing proteins, most importantly RIPK3 in the canonical necroptosis pathway. The DD mediates interactions with death-domain-containing adaptors and receptors and contributes to RIPK1 recruitment, dimerization, and activation within TNFR1-associated signaling complexes [13,14].

This domain organization explains why RIPK1 can behave as either a scaffold or a kinase. This ability to switch between scaffold-dependent inflammatory signaling and kinase-dependent cell death has positioned RIPK1 as a critical regulatory checkpoint in immune and stress responses. In receptor-proximal complexes, RIPK1 can serve as a ubiquitinated platform that recruits signaling proteins required for NF-kB and MAPK activation. The assembly of this signaling scaffold is tightly regulated by ubiquitin-modifying enzymes, which determine the stability and functional outcome of RIPK1-dependent complexes. Moreover, alterations in RIPK1 ubiquitination or complex composition can shift cellular responses from pro-survival signaling toward inflammatory or cell death pathways, including apoptosis and necroptosis. In cytosolic death-inducing complexes, the kinase domain can promote RIPK1 autophosphorylation, FADD/caspase-8 activation, or RHIM-mediated RIPK3 engagement. These molecular interactions determine the assembly and activity of distinct signaling platforms that regulate the balance between cell survival, apoptosis, and necroptotic cell death. Furthermore, the transition of RIPK1 from a scaffold function to a kinase-dependent pro-death mediator is controlled by multiple post-translational modifications and cellular context-dependent signals, ensuring precise regulation of inflammatory and immune responses. Specifically, loss of RIPK1 ubiquitination, deubiquitination by enzymes such as CYLD or A20, and specific phosphorylation events can destabilize receptor-proximal survival complexes and promote the formation of cytosolic death-inducing platforms [17,18]. Therefore, the most useful mechanistic distinction for AD is not simply RIPK1 “on” versus “off,” but whether RIPK1 operates mainly as a scaffold in inflammatory-survival signaling or as an active kinase in inflammatory cell-death signaling [13,19,20].

### 2.2. RIPK1 in Receptor-Proximal Inflammatory Signaling: TNFR1 Complex I

TNFR1 signaling provides the clearest framework for understanding RIPK1. After TNF-α binds TNFR1, the receptor recruits TRADD, RIPK1, TRAF2, cIAP1/2, and LUBAC to form membrane-associated complex I. Within this complex, cIAP1/2 and LUBAC generate K63-linked and M1-linked ubiquitin chains on RIPK1 and other complex I components. These ubiquitin chains create docking sites for TAK1/TAB and IKK/NEMO complexes, leading to MAPK activation, IKK activation, IkB degradation, NF-kB nuclear translocation, and transcription of cytokines and survival genes [21,22,23].

This signaling mode depends primarily on RIPK1 as a scaffold rather than on RIPK1 catalytic activity. This point is important because older descriptions sometimes imply that RIPK1 kinase activity directly drives canonical NF-kB activation. A more accurate model is that ubiquitinated RIPK1 helps organize complex I and supports inflammatory-survival signaling, whereas RIPK1 kinase activation becomes prominent when ubiquitin-dependent, phosphorylation-dependent, or caspase-dependent checkpoints fail. The prevention of cell death is ensured by maintaining RIPK1 in a ubiquitinated and kinase-restrained state, thereby inhibiting the formation of cytosolic death-inducing complexes and limiting inappropriate activation of caspase-8 or RIPK3-dependent necroptosis. Therefore, the balance between RIPK1 scaffold activity and kinase activation represents a critical regulatory checkpoint that determines whether cells undergo inflammatory adaptation, apoptosis, or necroptotic death depending on the cellular environment and the intensity of the stimulus. Thus, RIPK1 can participate in cytokine production and cell-state changes without committing the cell to apoptosis or necroptosis [13,24,25].

In the AD brain, this scaffold-dependent logic is particularly relevant to microglia and astrocytes. Plaques, damaged synapses, DAMPs, and cytokines can sustain TNF/TNFR1 and pattern-recognition receptor signaling. Under these conditions, RIPK1 may help stabilize inflammatory programs, including microglial disease-associated states, without necessarily activating full necroptosis. This concept is supported by evidence that RIPK1 inhibition or genetic manipulation modulates disease-associated microglial responses, lysosomal dysfunction, Aβ burden, and memory impairment in AD models [15,16].

### 2.3. Ubiquitin and Phosphorylation Checkpoints Restrain Cytotoxic RIPK1

RIPK1 activity is constrained by multiple checkpoints that prevent a physiological inflammatory response from becoming cytotoxic. Ubiquitination forms the first layer of control. cIAP1/2-mediated ubiquitination promotes complex I assembly and restrains death-complex formation, while specific ubiquitin linkages also directly limit RIPK1 kinase activation. Conversely, loss of cIAP activity, altered ubiquitin editing, or destabilization of complex I can shift RIPK1 away from receptor-proximal signaling and toward cytosolic death complexes [24,26,27].

Deubiquitinases add an extra layer of regulation. CYLD can remove ubiquitin chains from RIPK1 and facilitate necrosome formation under permissive conditions, whereas A20 can protect cells from TNF-induced death by stabilizing the linear ubiquitin network in complex I and opposing CYLD-dependent dismantling of that network. The A20/CYLD balance is therefore best viewed not as a simple on/off switch, but as part of a dynamic ubiquitin-editing system that determines whether RIPK1 remains in a pro-survival signaling complex or enters a cytotoxic complex [28,29].

Phosphorylation provides a second set of brakes. TAK1, IKKalpha/beta, and p38/MK2 can phosphorylate RIPK1 and restrain its cytotoxic activity during TNF signaling. IKK-mediated phosphorylation, including phosphorylation at Ser25, directly inhibits RIPK1 kinase-dependent death. MK2 phosphorylates conserved sites in the intermediate region of RIPK1 and suppresses TNF-induced RIPK1 activation. These phosphorylation events are conceptually important because they show that inflammatory signaling pathways do not simply activate RIPK1; they also actively prevent RIPK1 from crossing into apoptosis or necroptosis [25,30,31,32,33].

Autophosphorylation marks the opposite transition. Upon stimulation by factors such as tumor necrosis factor (TNF), RIPK1 is recruited to receptor signaling complexes where, under conditions that favor cell death rather than pro-survival signaling, it becomes activated through autophosphorylation at specific serine residues, particularly Ser166, which is widely used as a marker of RIPK1 kinase activation. This phosphorylation enhances RIPK1’s catalytic activity and promotes the formation of cytosolic death-inducing complexes, including complex II and the necrosome, through interactions with proteins such as RIPK3 and MLKL. Activated RIPK1 can therefore drive apoptosis or necroptosis depending on the cellular context and the availability of downstream effectors. In the context of AD, pS166-RIPK1 should therefore be interpreted as evidence of kinase activation rather than as a generic marker of RIPK1 expression. This distinction will matter for future biomarker studies and for trials of RIPK1 kinase inhibitors, because total RIPK1 levels and activated RIPK1 may report different biological states [13,14].

### 2.4. Caspase-8 Cleavage and the Apoptosis-Necroptosis Decision

Caspase-8 provides a final checkpoint that suppresses necroptosis while enabling apoptotic execution. In humans, caspase-8 cleaves RIPK1 after Asp324; in mice, the corresponding cleavage occurs after Asp325. This cleavage separates the N-terminal kinase domain from the intermediate region and DD, thereby limiting RIPK1 activation, dismantling death-inducing complexes, and preventing excessive apoptosis or necroptosis. Genetic evidence from non-cleavable RIPK1 models and human autoinflammatory disease supports the physiological importance of this checkpoint [34,35,36].

When caspase-8 is active, RIPK1 can contribute to RIPK1-dependent apoptosis through FADD/caspase-8-containing cytosolic complexes. When caspase-8 activity is absent, inhibited, or insufficient, RIPK1 can instead engage RIPK3 through RHIM-RHIM interactions. RIPK3 then phosphorylates MLKL, causing MLKL oligomerization, membrane association, membrane damage, and lytic cell death. Because necroptosis releases intracellular DAMPs, it can amplify inflammation in neighboring cells and create a feed-forward loop between cell death and innate immune activation [14,37,38].

### 2.5. RIPK1 Molecular Checkpoints and Their Relevance to Alzheimer’s Disease

The scaffold-versus-kinase distinction provides a framework for interpreting the role of RIPK1 in AD. In microglia and astrocytes, RIPK1 may help sustain inflammatory transcriptional programs downstream of TNF, DAMPs, Aβ-associated pattern-recognition signals, and other inflammatory triggers. In neurons or in glial cells exposed to stronger stress, impaired proteostasis, mitochondrial dysfunction, oxidative injury, or insufficient caspase-8 activity may lower the threshold for RIPK1 kinase-dependent apoptosis or RIPK1-RIPK3-MLKL necroptosis. These mechanisms are not mutually exclusive; they may occur in different cells or at different disease stages [14,16,39].

This model also provides insight into the potential therapeutic effects and limitations of RIPK1 inhibitors. A RIPK1 kinase inhibitor should suppress RIPK1 kinase-dependent apoptosis, necroptosis, and some kinase-dependent inflammatory outputs. However, it may not eliminate all RIPK1 scaffold-dependent signaling because RIPK1 retains kinase-independent scaffold activity that can sustain signaling pathways through adaptor functions and molecular interactions. Therefore, the therapeutic question in AD is not simply whether RIPK1 is increased, but which RIPK1 output is active, in which cell type, and at which disease stage. This is why RIPK1 should be framed as a context-dependent signaling checkpoint rather than a single linear necroptosis marker.

## 3. Evidence That RIPK1 Signaling Is Engaged in Alzheimer’s Disease

Evidence linking RIPK1 signaling to AD now comes from several experimental levels: human postmortem tissue, transcriptomic and genetic datasets, AD mouse models, human induced pluripotent stem cell (iPSC)-derived neurons, primary cultures, and pathway-based cellular systems (Table 1). These lines of evidence are complementary but not interchangeable. Human tissue establishes disease relevance, anatomical localization, and association with neuropathological severity. Animal and cellular systems provide stronger tests of causality and mechanism. Together, they support the idea that RIPK1 is not merely a marker of terminal injury, but a disease-relevant signaling node that may influence glial activation, inflammatory amplification, vascular dysfunction, and regulated neuronal death.

Throughout this review, we distinguish direct evidence of RIPK1 dependence from evidence of necroptosis-pathway activation more broadly. We consider evidence for RIPK1 dependence strongest when investigators directly manipulate RIPK1 genetically or pharmacologically and demonstrate an effect on the relevant phenotype, particularly when intervention specificity and target engagement are established. Studies that demonstrate RIPK3–MLKL activation, necroptosis, or changes in RIPK1-pathway markers without directly interrogating RIPK1 provide evidence of pathway engagement but do not establish RIPK1-specific causality. Similarly, unless experimental approaches directly distinguish RIPK1 kinase activity from its scaffold function, we do not assign observed AD-related effects specifically to either mechanism and instead treat such interpretations as mechanistic hypotheses. We therefore evaluate the evidence according to study type, causal strength, intervention specificity, evidence of target engagement, and direct relevance to AD.

### 3.1. Evidence from Human Postmortem and Omics Experiments

The strongest human evidence comes from studies that directly measured necroptosis-pathway components in AD brain tissue. We reported activation of the RIPK1-RIPK3-MLKL pathway in postmortem AD brains and showed that necroptosis markers correlated positively with Braak stage and inversely with brain weight and cognitive performance [16]. This study was important because it moved the field beyond the generic observation that neurons die in AD and linked neuronal loss to a specific regulated necrotic pathway. However, the data remain fundamentally associative: postmortem activation of RIPK1-pathway markers shows that the pathway is engaged in diseased tissue, but it cannot by itself determine whether RIPK1 initiates degeneration, amplifies injury, or marks vulnerable cells already committed to death.

A second line of human evidence localizes activated necroptosis machinery to granulovacuolar degeneration (GVD), a neuronal lesion strongly associated with AD and tau pathology. Koper et al. detected phosphorylated RIPK1, phosphorylated RIPK3, and phosphorylated MLKL in GVD bodies and found that necrosome-positive GVD was associated with neuronal loss in vulnerable brain regions, including CA1 and frontal cortex layer III [40]. This anatomical resolution matters because it places necroptosis-related proteins within neuronal compartments affected by AD rather than only in bulk tissue homogenates. Subsequent work showed that limbic-predominant age-related TDP-43 encephalopathy neuropathologic change can aggravate GVD-mediated necroptosis in AD, suggesting that comorbid proteinopathies may intensify or modify RIPK1-linked death pathways [46]. These observations support a model in which RIPK1-pathway activation may be particularly relevant in late, tau-associated, or mixed-pathology stages of disease.

Human hippocampal studies also connect the TNF inflammatory axis to neuronal necroptosis. Jayaraman et al. reported that TNF-mediated neuroinflammation is linked to neuronal necroptosis in the AD hippocampus and extended these findings to human iPSC-derived neurons exposed to TNF [41]. This work is useful for the present review because it helps bridge inflammation and regulated cell death: TNF signaling can engage RIPK1-dependent complexes, but the downstream outcome depends on the balance among inflammatory survival signaling, caspase-8 activity, RIPK3 availability, MLKL activation, and membrane repair pathways. Therefore, the human evidence supports a TNF–RIPK1–necroptosis axis, but it also emphasizes that RIPK1 activation must be interpreted within a broader inflammatory and cell-fate context.

Human evidence also implicates RIPK1 in glial pathology. Ofengeim et al. found that RIPK1 is highly expressed in microglia in human AD brains and used mouse models to show that RIPK1 promotes a disease-associated microglial program with lysosomal dysfunction and impaired Aβ handling [15]. This observation is central because it broadens the AD role of RIPK1 beyond neuronal necroptosis. In this framework, RIPK1 may regulate a pathogenic microglial state without necessarily killing the microglial cell. This distinction is critical for the whole review: RIPK1-dependent inflammatory reprogramming and RIPK1-dependent necroptosis overlap mechanistically, but they are not identical biological outputs.

Genetic and single-cell studies provide an important but more indirect context. Large-scale genetic studies have repeatedly implicated immune, microglial, lipid-handling, and inflammatory pathways in late-onset AD. More recent genetic analyses also highlight TNF-related and ubiquitin-regulatory biology, including the linear ubiquitin chain assembly complex, which is relevant to receptor-proximal RIPK1 regulation [7]. Single-cell and single-nucleus transcriptomic studies support a multicellular model of AD, in which vulnerable neurons, activated microglia, astrocytes, oligodendrocyte-lineage cells, and vascular-associated cells change in coordinated but cell-type-specific ways [12]. These omics studies do not prove RIPK1 activation by themselves. Rather, they justify asking whether RIPK1 sits within a broader disease network involving TNF signaling, ubiquitin checkpoints, microglial state transitions, and neuronal vulnerability.

### 3.2. Evidence from AD Animal Models

Animal studies provide the strongest evidence that RIPK1 signaling can modify AD-like phenotypes. In APP/PS1 mice, pharmacological and genetic inhibition of RIPK1 reduced Aβ burden, inflammatory cytokines, and memory deficits [15,16]. Mechanistically, this work linked RIPK1 to disease-associated microglia, lysosomal abnormalities, and impaired plaque handling. The major implication is that RIPK1 can act upstream of microglial dysfunction and Aβ accumulation, not only downstream of terminal neuronal death. This makes RIPK1 especially attractive as a disease-modifying target because a glial signaling mechanism could, in principle, alter the inflammatory environment before extensive neuronal loss occurs.

Studies in Aβ-driven models also support a role for RIPK1-RIPK3-MLKL necroptosis in cognitive and neurodegenerative outcomes. We showed that necroptosis-pathway activation occurs in AD models and that inhibition of the pathway can improve disease-relevant outcomes [16]. Salvadores et al. further demonstrated that Aβ oligomers can trigger neurodegeneration through a microglia-dependent necroptosis mechanism: conditioned medium from Aβ oligomer-stimulated microglia induced neuronal necroptosis through TNF-α signaling, whereas pharmacological or genetic inhibition of necroptosis reduced neurodegeneration and memory impairment in mice [44]. Together, these studies support a non-cell-autonomous model in which Aβ pathology activates microglia, microglia release inflammatory mediators such as TNF-α, and neurons then engage RIPK1-linked death programs.

Tau-related models strengthen the case that RIPK1-linked necroptosis is not limited to Aβ pathology. Dong et al. reported that hyperphosphorylated tau can induce neuronal necroptosis and inflammation through the RIPK1-RIPK3-MLKL axis and that necrostatin (Nec-1s) treatment mitigated tau-associated inflammatory and neuronal phenotypes in a tauopathy model [45]. Koper et al. later described an AD-associated form of GVD-necroptosis in mouse models and primary neurons, with stronger activation in tau-pathology settings than in Aβ-only settings; inhibition of necroptosis reduced GVD and neuronal loss [49]. This distinction is important for the review because it suggests that RIPK1-mediated neuronal death may become especially prominent when tau pathology, GVD, and neuronal stress pathways converge.

Additional model systems expand the pathogenic scope of RIPK1 signaling. Xu et al. described TNF-α-dependent neuronal necroptosis in AD that involves coordination between a RIPK1-p62 complex and the autophagic regulator UVRAG, linking inflammatory death signaling to autophagy control [42]. Park et al. reported that increasing O-GlcNAcylation ameliorated AD-related pathology while inhibiting necroptosis, placing RIPK1-RIPK3-MLKL signaling within a broader metabolic and post-translational regulatory network [43]. Zou et al. extended the pathway to the neurovascular unit by showing that reduced mNAT1/hNAT2 contributes to cerebral endothelial necroptosis and Aβ accumulation [47]. These findings support the view that RIPK1-linked mechanisms may affect several AD-relevant compartments, including neurons, microglia, and endothelial cells.

### 3.3. Evidence from Cellular Systems

Cellular systems provide mechanistic resolution that human tissue and whole-animal models cannot easily achieve. In neuron–microglia paradigms, Aβ oligomer-stimulated microglia can induce neuronal necroptosis through soluble inflammatory mediators, particularly TNF-α [44]. This type of experiment separates the cell that senses Aβ from the cell that dies, and it directly supports the idea that RIPK1 signaling can mediate non-cell-autonomous neurotoxicity. It also fits with the broader AD literature in which microglia can shift from protective plaque-containment and debris-clearance functions toward inflammatory states that damage synapses and neurons [52].

Human iPSC-derived neuronal systems add translational value because they avoid some species-specific limitations of rodent neurons. Jayaraman et al. used human iPSC-derived neurons to test TNF-linked neuronal necroptosis and found evidence that necroptosis-associated stress interacts with membrane repair mechanisms, including ESCRT-III components [41]. These findings support a model in which neurons may not passively undergo immediate lysis after MLKL activation; instead, the outcome may depend on the balance between necroptotic membrane injury and repair pathways. This point is relevant for AD, where slowly progressive degeneration could reflect chronic sublethal pathway activation, delayed cell death, or repeated inflammatory stress rather than an acute necroptotic burst.

Tau- and TNF-based cellular models provide additional mechanistic entry points. Hyperphosphorylated tau can activate RIPK1-RIPK3-MLKL signaling in neuronal systems, whereas TNF-α can engage RIPK1-dependent death signaling when protective checkpoints are overwhelmed or disabled [42,45]. Endothelial cell models similarly suggest that RIPK1-linked necroptosis can alter vascular integrity and Aβ accumulation [47]. These models are reductionist by design, but they help identify testable mechanisms: inflammatory receptor activation, impaired autophagy, tau-associated stress, altered metabolism, and defective membrane repair may all lower the threshold for RIPK1-dependent injury.

### 3.4. Strength of Evidence and Limitations

Cumulative evidence supports RIPK1 as a disease-relevant pathway in AD, but the strength of the evidence differs by claim. The strongest human evidence shows that components of the RIPK1–RIPK3–MLKL/necroptosis pathway are activated in AD brain and are associated with disease severity, neuronal lesions, and neuroinflammatory state. Multiple studies detect RIPK1, RIPK3, MLKL, or their activated forms in AD tissue, and several connect these markers to Braak stage, cognitive decline, neuronal loss, GVD, or TNF-linked inflammation. A second strong claim is that manipulating RIPK1 or downstream necroptosis machinery can modify AD-relevant phenotypes in experimental models [15,16,40,44].

Independent studies have reproduced several of these broad observations, although replication remains uneven for specific mechanisms. Distinct human cohorts and experimental approaches have consistently identified necroptosis-related pathway engagement in AD brain, and independent experimental studies have linked tau-associated pathology to RIPK1–RIPK3–MLKL/necroptosis signaling. By contrast, more specific mechanisms, including RIPK1-dependent disease-associated microglial reprogramming, MEG3-mediated human-neuron necroptosis, and endothelial NAT1-associated necroptosis, currently rely more heavily on individual studies and require independent replication.

The weaker claim would be that RIPK1 is already proven to be a primary causal driver of human AD. The field has not yet reached that point. Postmortem studies are vulnerable to confounding by cell loss, agonal state, disease stage, and comorbid pathologies, as well as pre-analytical variables such as postmortem interval, fixation, storage conditions, and tissue handling. These factors can affect protein preservation and phosphorylation-dependent measurements. Bulk-tissue analyses also cannot reliably assign pathway activation to specific cell types. In addition, antibody-based detection of phosphorylated RIPK1, RIPK3, or MLKL requires rigorous validation, particularly in aged human tissue. Importantly, detection of these activated pathway components demonstrates engagement of necroptosis-related signaling but does not by itself establish that a cell completed necroptotic death. Demonstrating completed necroptosis requires evidence that pathway activation progressed to terminal membrane-disruptive cell death rather than representing transient, incomplete, or compartmentalized signaling. Pharmacological studies also require caution because older inhibitors can have off-target effects, and many experiments do not fully distinguish RIPK1 kinase-dependent effects from RIPK1 scaffold-dependent inflammatory functions.

A balanced interpretation is therefore that RIPK1 signaling is engaged in AD and can plausibly contribute to several disease mechanisms, but its exact role is likely stage- and cell-type-dependent. In microglia, RIPK1 may promote inflammatory reprogramming, lysosomal dysfunction, and impaired Aβ handling. In neurons, RIPK1 may help execute or amplify necroptosis, particularly under TNF-rich, tau-associated, or autophagy-impaired conditions. In vascular cells, RIPK1-linked necroptosis may contribute to blood–brain barrier dysfunction and Aβ accumulation. Thus, the most useful working model is not that RIPK1 alone causes AD, but that RIPK1 signaling may serve as an understudied inflammatory and cell-death checkpoint that connects several AD pathogenic processes.

## 4. Cell-Type-Specific Roles of RIPK1 in the AD Brain

A cell-type-specific framework provides the clearest way to interpret RIPK1 biology in AD. RIPK1 signaling does not produce a single uniform output across the brain. Instead, each cell type interprets RIPK1 activation according to its receptor repertoire, ubiquitin-editing machinery, caspase-8 status, RIPK3 and MLKL expression, metabolic state, and exposure to Aβ, tau, cytokines, and oxidative stress. This distinction matters because the same pathway can support inflammatory transcription in one cell type, regulated cell death in another, and non-cell-autonomous tissue injury through communication between glia, neurons, and vascular cells.

The available evidence supports a working model in which RIPK1 acts primarily as an inflammatory and state-transition regulator in microglia, a potential amplifier of reactive and neurotoxic programs in astrocytes, a mediator of regulated death in vulnerable neurons, and an emerging contributor to oligodendroglial and vascular dysfunction. This model remains provisional. Direct AD-specific evidence is strongest for microglia, neurons, and endothelial cells, whereas astrocytic and oligodendroglial roles require more focused testing.

### 4.1. Microglia: RIPK1 as a Regulator of Inflammatory and Disease-Associated States

Microglia represent the strongest cell-type-specific link between RIPK1 and AD pathogenesis. During Aβ accumulation, microglia move away from homeostatic programs and enter plaque-associated and disease-associated states. Single-cell work in AD models identified disease-associated microglia (DAM), which localize near Aβ plaques and show altered phagocytic, lipid-handling, inflammatory, and lysosomal programs [10]. These states should not be viewed as purely harmful. Early microglial activation can restrict plaque toxicity and promote containment. However, prolonged or dysregulated microglial activation can impair clearance, increase cytokine production, and amplify neuronal injury.

RIPK1 provides a mechanistic entry point into this maladaptive microglial biology. Ofengeim et al. showed that RIPK1 promotes a disease-associated microglial response in AD, including transcriptional changes and lysosomal dysfunction linked to plaque accumulation [15]. In APP/PS1 mice, RIPK1 inhibition reduced inflammatory and DAM-related features, supporting the idea that RIPK1 contributes to microglial reprogramming rather than merely marking activated microglia. This finding is important because it places RIPK1 upstream of glial state changes that may influence Aβ handling and inflammatory tone.

Microglial RIPK1 may also drive neuronal injury indirectly. Aβ oligomers activate microglia and promote TNF-dependent signaling that can trigger neuronal necroptosis. Salvadores et al. showed that conditioned medium from Aβ oligomer-stimulated microglia induced neuronal necroptosis through TNF-α signaling, and that pharmacological or genetic necroptosis inhibition protected neurons and improved Aβ oligomer-induced outcomes [44]. This supports a non-cell-autonomous model: microglial RIPK1-associated inflammatory signaling may sensitize nearby neurons to RIPK1-RIPK3-MLKL death signaling, even when the initiating stimulus originates from Aβ.

RIPK1 should therefore be considered a regulator of microglial function, not simply a microglial death protein. In microglia, RIPK1 can participate in inflammatory transcription, lysosomal dysfunction, cytokine release, and, under specific checkpoint conditions, apoptosis or necroptosis. The key unresolved question is which of these outputs dominates in the AD brain. Conditional RIPK1 manipulation in microglia, combined with spatial transcriptomics and phospho-RIPK1/RIPK3/MLKL mapping, will be necessary to determine whether RIPK1 mainly sustains maladaptive microglial states, causes microglial loss, or couples both processes.

### 4.2. Astrocytes: Inflammatory Amplification and Loss of Homeostatic Support

Astrocytes are central regulators of synaptic homeostasis, extracellular glutamate, ion balance, metabolic support, lipid handling, and blood–brain barrier function. In AD, astrocytes undergo heterogeneous reactive changes that vary by brain region, disease stage, and proximity to pathology [12,53]. These transcriptional changes make astrocytes plausible contributors to RIPK1-dependent pathology. However, direct AD-specific evidence for an astrocytic RIPK1 mechanism remains limited. The mechanistic possibilities discussed below should therefore be considered indirect or hypothesis-generating rather than established features of AD.

Two lines of evidence nevertheless justify including astrocytes in a RIPK1-centered AD model. First, activated microglia can induce neurotoxic reactive astrocyte states through IL-1alpha, TNF, and C1q [54]. Because RIPK1 can regulate inflammatory signaling and cytokine production in microglia, RIPK1 may indirectly promote astrocyte reactivity through microglia–astrocyte crosstalk. Second, work in inflammatory CNS disease shows that astrocytes can use RIPK1 kinase activity to drive inflammatory transcription without necessarily undergoing necroptosis. Zelic et al. found that RIPK1 activation in astrocytes produced inflammatory gene-expression programs and non-cell-autonomous effects, whereas astrocytes were relatively resistant to RIPK1-mediated cell death compared with microglia [55]. Although this study focused on multiple sclerosis rather than AD, it provides a useful mechanistic principle: RIPK1 activation can promote glial inflammation even when it does not culminate in lytic cell death.

In AD, this principle suggests that astrocytic RIPK1 could contribute to disease by amplifying cytokine and chemokine responses, altering complement signaling, reducing support functions, or modifying lipid and metabolic pathways. However, these possibilities remain hypotheses until tested directly. The field needs astrocyte-specific Ripk1 genetic models in Aβ and tau contexts, ideally combined with functional readouts such as glutamate uptake, synapse maintenance, metabolic support, and microglia–astrocyte ligand-receptor interactions.

### 4.3. Neurons: RIPK1-Dependent Death Signaling and Vulnerability to Aβ, Tau, and TNF

Neurons appear most relevant to RIPK1-dependent regulated cell death. Multiple studies have reported activation of the RIPK1-RIPK3-MLKL pathway in human AD tissue and AD-related models. We showed that necroptosis markers are increased in postmortem AD brains, correlate with Braak stage, and inversely correlate with brain weight and cognitive scores [16]. Koper et al. further localized activated necrosome components to granulovacuolar degeneration, a neuronal lesion associated with AD and neuronal loss [40]. Together, these studies place RIPK1-linked necroptosis machinery in vulnerable neuronal compartments and connect it to disease severity.

Inflammatory and proteopathic triggers can both feed into neuronal RIPK1 signaling. Jayaraman et al. linked TNF-mediated neuroinflammation to neuronal necroptosis in the AD hippocampus and supported this mechanism using human iPSC-derived neurons [41]. Dong et al. reported that hyperphosphorylated tau can induce necroptosis and inflammatory signaling through the RIPK1-RIPK3-MLKL axis [45]. More recently, human-neuron xenograft work showed that Aβ pathology can produce a human-specific neuronal vulnerability involving the long noncoding RNA MEG3 and necroptosis; genetic or pharmacological manipulation of RIPK1, RIPK3, or MLKL rescued neuronal loss in this model [48].

These findings support neuronal RIPK1 signaling as a plausible effector of degeneration, but they also require careful interpretation. Postmortem detection of pRIPK1, pRIPK3, or pMLKL does not by itself prove that necroptosis caused neuronal death. Neurons may activate incomplete or abortive death programs, and necroptosis markers may accumulate in damaged compartments without always leading to rapid lysis. Therefore, the most defensible conclusion is that neuronal RIPK1-RIPK3-MLKL signaling is engaged in AD and can drive neuronal loss in experimental systems, but its quantitative contribution to neuronal death across human AD stages remains unresolved.

### 4.4. Oligodendrocytes and White Matter: An Emerging but Underdeveloped AD Area

Oligodendrocytes and white matter changes remain understudied in RIPK1-focused AD research. This is a gap because white matter injury, myelin dysfunction, and oligodendrocyte stress occur in aging and AD, and single-nucleus studies have revealed subtype-specific oligodendroglial and astrocytic changes in AD brains [53]. However, direct evidence that RIPK1 drives oligodendrocyte pathology in AD is currently lacking. The rationale for considering such a role derives largely from studies in other inflammatory CNS disorders and should therefore be viewed as hypothesis-generating.

Evidence from other CNS diseases suggests that this question deserves attention. In multiple sclerosis models and tissue, RIPK1-linked necroptosis and inflammatory signaling can affect oligodendrocytes and myelination. Ofengeim et al. showed that RIPK1 inhibition protects oligodendrocytes from TNF-induced necroptosis in an MS context [39]. Zelic et al. later showed that RIPK1 activation in microglia and astrocytes can produce non-cell-autonomous effects that impair oligodendrocyte precursor cells and myelination [55]. These findings should not be transferred uncritically to AD, but they provide a rationale for testing whether AD-associated inflammatory environments activate similar RIPK1-dependent glia-oligodendrocyte injury programs.

For AD, the most likely role of RIPK1 in oligodendroglial biology may be indirect. Microglial and astrocytic RIPK1 activation could create a cytokine-rich, oxidative, and metabolically unfavorable environment that compromises oligodendrocyte maturation and myelin maintenance. Future studies should determine whether RIPK1 inhibition preserves white matter integrity or oligodendrocyte states in Aβ and tau models, especially during aging, when myelin repair capacity declines.

### 4.5. Endothelial Cells and the Neurovascular Unit

The neurovascular unit provides another important and more directly AD-relevant context for RIPK1 signaling. Cerebrovascular dysfunction and blood–brain barrier disruption can impair Aβ clearance, promote inflammatory entry points, and worsen neuronal vulnerability. Zou et al. identified venular and capillary endothelial cells as selectively vulnerable to necroptosis in AD. They reported reduced mNat1 in AD mouse models and reduced hNAT2 in human AD, and showed that mNat1 deficiency promotes blood–brain barrier damage, endothelial necroptosis, A20 degradation, LRP1beta loss, Aβ accumulation, and cognitive impairment [47]. Conversely, endothelial restoration of mNAT1 rescued A20 and LRP1beta, inhibited endothelial necroptosis and activation, reduced Aβ deposits, and improved cognition in an AD mouse model.

This vascular work expands the RIPK1 model beyond neurons and glia. It suggests that RIPK1-related necroptosis may compromise barrier integrity and Aβ export, thereby converting vascular dysfunction into a feed-forward driver of Aβ accumulation and inflammation. Because endothelial cells also interact with astrocytic endfeet, pericytes, microglia, and circulating immune mediators, vascular RIPK1 activation could influence several AD-relevant compartments simultaneously. The vascular arm of RIPK1 biology may therefore be particularly relevant for sporadic AD, where metabolic dysfunction, insulin resistance, vascular disease, and aging often coexist with Aβ and tau pathology.

### 4.6. Integrative Model: RIPK1 as a Multicellular Signaling Checkpoint

**Figure 2:**
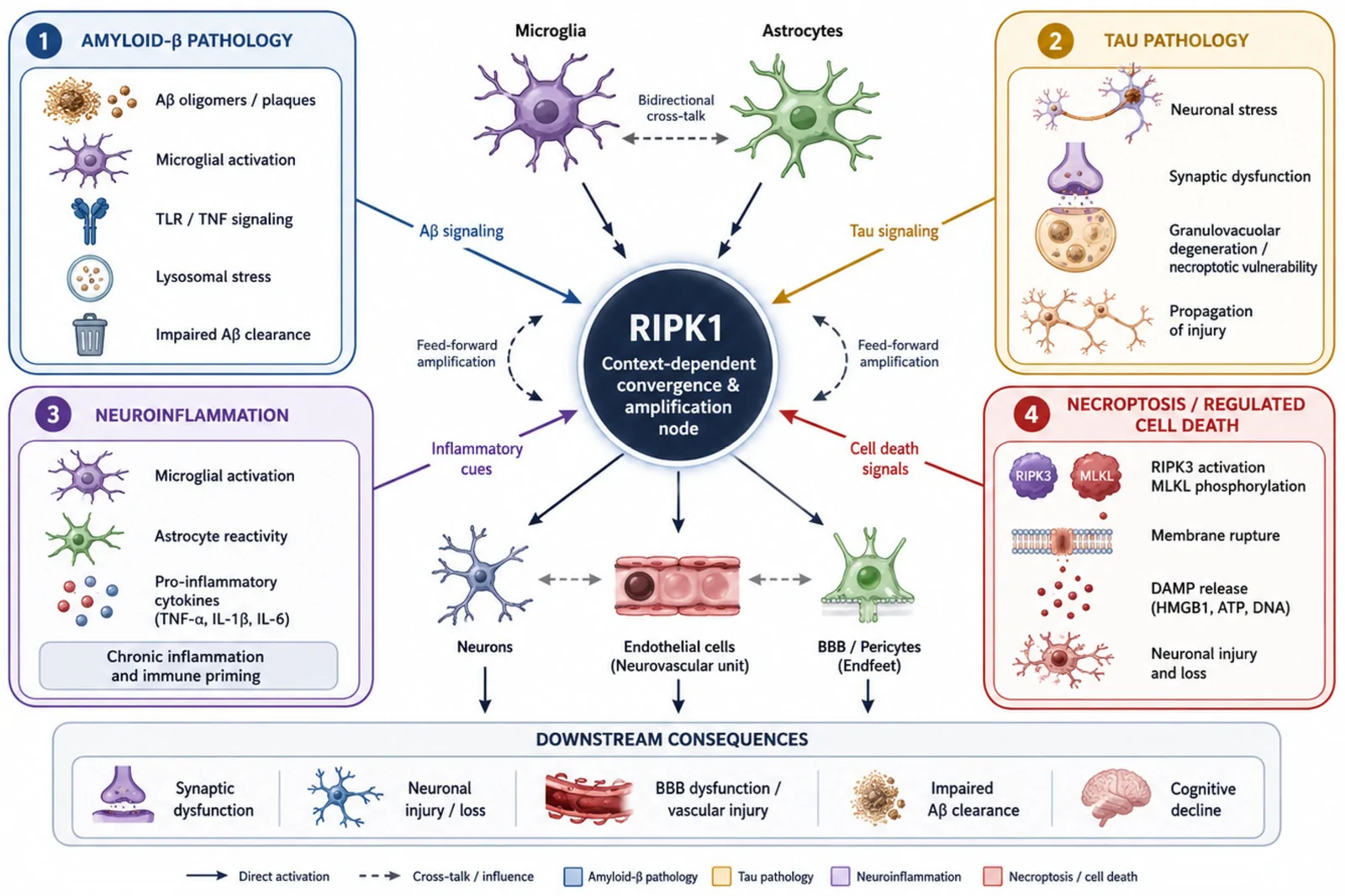
RIPK1 as a context-dependent convergence node in Alzheimer’s disease. RIPK1 may integrate signals from Aβ pathology, tau-mediated neuronal stress, neuroinflammation, and regulated cell death across multiple CNS cell types. In this model, Aβ, tau, inflammatory cytokines, glial activation, and necroptotic signaling converge on RIPK1-dependent pathways that amplify synaptic dysfunction, neuronal injury, impaired Aβ clearance, blood–brain barrier dysfunction, and cognitive decline. Rather than acting as an initiating cause of Alzheimer’s disease, RIPK1 may function as a context-dependent amplifier of interacting pathogenic processes. The diagram represents a conceptual framework, and the depicted relationships do not all have equivalent evidentiary support; the strength and directness of the underlying evidence are detailed in Table 1 and the accompanying text.

Taken together, the available evidence argues against a uniform model in which RIPK1 produces the same biological outcome across all AD-relevant cell types. Direct AD-specific evidence is strongest for microglial inflammatory reprogramming, neuronal RIPK1–RIPK3–MLKL signaling, and endothelial necroptosis, whereas proposed astrocytic and oligodendroglial functions remain less directly supported. These cell-type differences provide the basis for the convergence model developed in the following section (Figure 2).

## 5. RIPK1 as a Convergence Point for Aβ, Tau, Inflammation, and Necroptosis

The evidence discussed above supports a model in which RIPK1 functions not as an initiating cause of AD, but as a context-dependent signaling checkpoint that may couple several pathogenic processes once disease is established. Aβ-associated glial activation, tau-mediated neuronal stress, chronic inflammatory signaling, and regulated cell death can each create conditions that alter RIPK1 activity or its downstream consequences. In turn, RIPK1-dependent inflammatory and death pathways may reinforce these processes, generating feed-forward interactions among glial dysfunction, neuronal vulnerability, vascular injury, and cell death (Figure 2). Importantly, this model does not require all AD-relevant effects of RIPK1 to arise through the same molecular mechanism. Scaffold-dependent signaling may predominate in some cellular contexts, whereas kinase activation and RIPK1–RIPK3–MLKL signaling may become more important when regulatory checkpoints fail [14,15,16].

### 5.1. A Working Mechanistic Model: RIPK1 as a Context-Dependent Amplifier

We propose a working model in which RIPK1 occupies an intermediate position between upstream AD pathology and downstream inflammatory and cytotoxic responses. In this framework, Aβ and tau provide distinct but interacting sources of cellular stress. Aβ accumulation can activate microglia and alter inflammatory and lysosomal programs, whereas tau pathology can increase neuronal vulnerability and associate with necrosome-positive degenerative lesions. Evidence for a reciprocal influence of RIPK1 signaling on tau pathology is suggestive but remains indirect. Pharmacological inhibition of RIPK1/necroptosis has been associated with reduced tau hyperphosphorylation in several experimental settings, including APP/PS1 mice and other AD-relevant models [16,56,57]. However, these studies do not establish that RIPK1 kinase activation directly promotes tau phosphorylation, and interpretation of Nec-1 studies is complicated by evidence that Nec-1 can also bind tau and inhibit its aggregation. Thus, current findings are compatible with bidirectional coupling between RIPK1 signaling and tau pathology, but direct evidence that RIPK1 drives tau hyperphosphorylation, tau seeding, or prion-like propagation remains lacking. Persistent cytokine signaling, particularly through TNF/TNFR1, provides a mechanistic route through which these disease-associated stresses can engage RIPK1-dependent signaling [15,16,41,42,44,45].

The cellular outcome should depend on both the cell type and the molecular state of RIPK1. In microglia, RIPK1 can promote disease-associated transcriptional programs, cytokine production, and lysosomal dysfunction, potentially altering Aβ handling without necessarily causing microglial death [15]. In vulnerable neurons, failure of protective checkpoints can instead favor kinase-dependent RIPK1 activation and downstream RIPK3–MLKL signaling, particularly in the presence of TNF signaling, tau-associated stress, or other conditions that lower the threshold for regulated cell death [16,41,42,44,45,48]. Endothelial necroptosis may provide an additional route through which RIPK1-related signaling contributes to blood–brain barrier dysfunction and impaired Aβ clearance, although the specific contribution of RIPK1 to this vascular mechanism requires further definition [47].

These responses could form a self-reinforcing circuit. Aβ-associated glial activation increases inflammatory signaling; persistent inflammation increases neuronal stress and can facilitate RIPK1-dependent death signaling; necroptotic cells release damage-associated molecular patterns that further stimulate innate immune responses; and vascular or lysosomal dysfunction may reduce the brain’s ability to contain or clear pathological proteins. RIPK1 would therefore function as an amplifier of pathological coupling rather than as a single linear mediator linking Aβ or tau directly to neuronal death.

### 5.2. Evidence-Supported Versus Provisional Connections

Several components of this model have direct experimental support. First, RIPK1-related necroptosis machinery is activated in human AD brain, and pathway markers associate with neuropathological severity, neuronal loss, and cognitive measures [16,40]. Second, genetic and pharmacological manipulation of RIPK1 modifies disease-associated microglial states, lysosomal dysfunction, Aβ burden, and behavioral phenotypes in AD models [15]. Third, Aβ oligomer-stimulated microglia can induce TNF-dependent neuronal necroptosis, providing experimental evidence for a non-cell-autonomous link between Aβ-associated glial activation and neuronal death [44]. Fourth, tau-related experimental systems show activation of RIPK1–RIPK3–MLKL signaling, and inhibition of this pathway reduces neuronal and inflammatory phenotypes [45,49]. Finally, human-neuron xenograft experiments demonstrate that an AD-like environment can induce necroptotic neuronal loss and that genetic or pharmacological inhibition of RIPK1 pathway components can rescue this phenotype [48].

Other elements of the convergence model remain provisional. Current evidence does not establish that Aβ or tau directly activates RIPK1 in human AD neurons, nor does it demonstrate that RIPK1 initiates either Aβ deposition or tau aggregation. Similarly, the relative contribution of RIPK1 scaffold activity and kinase activity to disease-associated microglial reprogramming remains unresolved. Human postmortem studies establish pathway engagement and anatomical association but cannot determine temporal order or causality. It also remains unclear whether inflammatory-glial signaling and neuronal necroptosis occur sequentially, simultaneously, or independently in different brain regions and disease stages.

The directionality of the proposed feedback loops is therefore a central unresolved issue. RIPK1 activation could actively convert protective inflammatory responses into maladaptive ones, or it could occur primarily after cells have already entered a dysfunctional state. Likewise, necroptosis could contribute substantially to inflammatory amplification, or it could represent one of several parallel consequences of advanced cellular stress. These alternatives are experimentally distinguishable and should not be treated as equivalent versions of the same model.

### 5.3. Testable Predictions of the Convergence Model

A useful convergence model should generate predictions that extend beyond the observations from which it was constructed. The first prediction is cell-type specificity. If RIPK1 serves different functions across CNS compartments, manipulating RIPK1 in microglia should preferentially alter inflammatory transcription, lysosomal function, cytokine signaling, and Aβ handling, whereas neuronal manipulation should have a stronger effect on RIPK1–RIPK3–MLKL activation and neuronal survival. Manipulation in endothelial cells should preferentially affect vascular integrity and Aβ clearance. Similar outcomes across all cell types would argue against the proposed compartment-specific model.

The second prediction concerns the distinction between scaffold-dependent and kinase-dependent RIPK1 functions. Selective inhibition of RIPK1 kinase activity should strongly suppress pS166-RIPK1 activation, downstream necroptosis signaling, and RIPK1-dependent neuronal death. However, kinase inhibition may not fully reproduce the effects of eliminating RIPK1 if disease-associated microglial transcriptional or lysosomal phenotypes depend partly on scaffold functions. Comparing kinase-selective inhibition or kinase-inactive RIPK1 with cell-specific RIPK1 depletion would therefore provide a direct test of the proposed two-output model.

Third, the amplifier model predicts a temporal relationship between established AD pathology and RIPK1 activation. RIPK1 pathway activation should increase as Aβ- or tau-associated cellular stress becomes accompanied by maladaptive inflammation, neuronal injury, or vascular dysfunction rather than uniformly preceding these pathologies. Longitudinal analyses should therefore identify disease stages or anatomical regions in which increased pS166-RIPK1, RIPK3/MLKL activation, or RIPK1-associated inflammatory programs coincide with the transition from relatively contained pathology to progressive tissue injury.

Finally, if necroptosis participates in a feed-forward inflammatory loop, blocking RIPK1–RIPK3–MLKL signaling should reduce not only neuronal death but also at least some downstream inflammatory consequences of cell lysis. Such an intervention need not eliminate the initiating Aβ or tau pathology to support the amplifier model. Conversely, substantial reductions in Aβ or tau pathology after RIPK1 manipulation would suggest that RIPK1 also influences upstream disease processes, as already suggested by the effects of RIPK1 manipulation on microglial function and Aβ burden.

### 5.4. Experimental Strategies That Could Validate or Disprove the Model

Several experimental approaches can distinguish these possibilities. First, cell-type-specific manipulation of RIPK1 should be combined with models that develop Aβ pathology, tau pathology, or both. Conditional deletion of RIPK1 in microglia, neurons, or endothelial cells, together with kinase-selective genetic or pharmacological inhibition, would separate cell-autonomous effects from non-cell-autonomous effects and help distinguish scaffold-dependent signaling from kinase-dependent cell death. Relevant outcomes should include microglial transcriptional states and lysosomal function, Aβ burden and clearance, pS166-RIPK1 and RIPK3–MLKL activation, neuronal loss, vascular integrity, and behavioral phenotypes.

Second, temporal and spatial mapping will be necessary to establish sequence rather than association. Longitudinal studies that integrate phospho-RIPK1/RIPK3/MLKL measurements with cell-type-resolved transcriptomic or spatial approaches could determine where and when RIPK1 activation emerges relative to Aβ deposition, tau pathology, glial-state transitions, and neuronal injury. Human tissue spanning neuropathological stages would provide complementary evidence, although cross-sectional postmortem material cannot independently establish temporal causality.

Third, human cellular systems can directly test the proposed glial-neuronal coupling. Human iPSC-derived neurons combined with microglia or microglial conditioned-medium paradigms can determine whether Aβ-associated glial signals are sufficient to lower the threshold for neuronal RIPK1 activation and whether disruption of TNF/TNFR1, RIPK1, RIPK3, or MLKL interrupts this sequence [41,44]. Human-neuron xenograft systems provide an additional opportunity to test whether pathway manipulation prevents neuronal loss within an AD-like brain environment [48].

These experiments also define conditions that would weaken or falsify important components of the model. The proposed role of RIPK1 as a causal amplifier would be difficult to sustain if cell-type-specific genetic disruption of RIPK1 failed to modify the predicted inflammatory, lysosomal, vascular, or neuronal phenotypes despite verified pathway manipulation. The proposed distinction between scaffold- and kinase-dependent outputs would be weakened if selective kinase inhibition consistently reproduced all effects of RIPK1 depletion, including disease-associated microglial transcriptional and lysosomal changes. Similarly, the feed-forward model would be challenged if suppression of RIPK1-dependent necroptosis reduced cell death without altering downstream inflammatory signaling, or if longitudinal analyses showed no spatial or temporal relationship between RIPK1 activation and transitions toward neuronal injury.

Thus, the key question is no longer simply whether RIPK1 signaling occurs in AD. Available evidence supports pathway engagement in human disease and functional effects in experimental systems, but it does not yet establish where RIPK1 sits in the causal hierarchy of AD pathogenesis. Distinguishing RIPK1 as a downstream marker, a context-dependent amplifier, or a therapeutically actionable convergence point will require experiments that resolve cell type, molecular function, and temporal sequence. This framework provides a testable basis for evaluating the therapeutic implications of RIPK1 inhibition discussed in the following section.

## 6. Therapeutic Targeting of RIPK1 in Alzheimer’s Disease: Promise, Caveats, and Translational Requirements

The therapeutic appeal of RIPK1 in AD derives from the same property that makes its biology complex: RIPK1 lies at the intersection of inflammatory signaling, cell-fate checkpoints, and tissue injury. Inhibiting RIPK1 therefore has the potential to modify more than one pathogenic component of AD. However, this therapeutic logic also creates a substantial translational challenge. A successful RIPK1-based intervention will require defining which RIPK1 output is being targeted, which CNS cell types contribute to disease progression, when the pathway is active, and whether pharmacological target engagement produces meaningful changes in AD-relevant biology.

For this reason, RIPK1 should not be framed simply as a necroptosis target. A more useful therapeutic model is that RIPK1 inhibition may attenuate a context-dependent disease program that includes maladaptive microglial activation, inflammatory cytokine signaling, lysosomal dysfunction, neuronal susceptibility to regulated cell death, and potentially neurovascular injury. This broader view supports therapeutic interest in RIPK1, but it also argues for biomarker-guided and stage-informed development rather than treatment of biologically unselected patients.

### 6.1. Rationale for Targeting RIPK1 in AD

Several observations provide the biological rationale for RIPK1 inhibition in AD. First, necroptosis markers are increased in postmortem AD brains and correlate with neuropathological and clinical measures, linking the RIPK1–RIPK3–MLKL axis to disease severity [16]. Second, RIPK1 promotes a disease-associated microglial response in AD models, including transcriptional changes and lysosomal dysfunction associated with Aβ plaque accumulation [15]. Third, Aβ oligomer-stimulated microglia can induce neuronal necroptosis through TNF-dependent mechanisms, placing RIPK1-linked glial inflammation upstream of neuronal death signaling [44]. Fourth, human-neuron xenograft experiments show that necroptosis can mediate neuronal vulnerability in an AD-like environment and that genetic or pharmacological inhibition of RIPK1 pathway components can rescue neuronal loss [48].

Together, these studies suggest that RIPK1 may act at both upstream and downstream levels of AD pathogenesis. Upstream, RIPK1 can influence glial inflammatory states and plaque-associated microglial dysfunction. Downstream, RIPK1 kinase activation can promote RIPK1-dependent apoptosis or RIPK1–RIPK3–MLKL necroptosis in vulnerable cells. The therapeutic rationale therefore rests on the possibility that RIPK1 inhibition could limit inflammatory amplification while also reducing regulated cell death. This dual mechanism is attractive, but it also means that clinical development must determine which component of RIPK1 biology is active and therapeutically relevant in a given patient population.

### 6.2. Pharmacological RIPK1 Inhibition: From Nec-1 to CNS-Penetrant Inhibitors

Necrostatin-1 (Nec-1) and related analogs were essential experimental tools for establishing RIPK1 kinase activity as a druggable component of necroptosis. However, these compounds should be interpreted cautiously in a therapeutic context. Original Nec-1 has off-target activity, including inhibition of indoleamine 2,3-dioxygenase, whereas Nec-1s is more stable and more selective for RIPK1 [58,59]. Thus, Nec-1-based preclinical findings establish pathway biology but do not by themselves demonstrate clinical druggability in AD.

The translational landscape changed with the development of orally bioavailable, brain-penetrant RIPK1 inhibitors. SAR443060 (DNL747) reached phase I/Ib studies in healthy participants and patients with AD or amyotrophic lateral sclerosis (ALS). Oral administration resulted in CSF exposure and robust peripheral target engagement, measured by suppression of RIPK1 phosphorylation at serine 166 (pS166-RIPK1) [50]. Treatment was generally well tolerated during the relatively short clinical exposure periods. These studies provided proof that pharmacological modulation of RIPK1 can be achieved in humans, including patients with neurodegenerative disease.

SAR443820 (DNL788; oditrasertib) subsequently provided further evidence for the feasibility of CNS-directed RIPK1 inhibition. In a first-in-human study in healthy adults, SAR443820 showed favorable pharmacokinetics, high CNS penetration, and strong pharmacodynamic target engagement. The mean CSF-to-unbound plasma concentration ratio ranged from approximately 0.8 to 1.3, and repeated administration produced close to 90% maximal median inhibition of pS166-RIPK1 in peripheral blood mononuclear cells [51]. Short-term administration was generally well tolerated, with no clinically meaningful laboratory, vital-sign, or electrocardiographic abnormalities reported [51]. These findings demonstrate CNS exposure and pharmacological target engagement but do not establish therapeutic efficacy in AD or other neurodegenerative disorders.

More recently, SIR9900 provided independent evidence that these pharmacological properties can be achieved with another brain-penetrant RIPK1 inhibitor. In a phase I study involving healthy adults and a small cohort of participants aged ≥65 years, SIR9900 showed a CSF-to-unbound plasma concentration ratio of 1.15 and approximately 90% peripheral target engagement, with sustained RIPK1 inhibition during repeated dosing [60]. Treatment was well tolerated over the short duration studied, and systemic exposure was similar in younger and elderly participants [60]. Although these findings remain early-phase data, they strengthen the evidence that CNS penetration and substantial RIPK1 kinase inhibition are pharmacologically achievable across distinct compounds.

### 6.3. What Published Clinical Studies Establish and What They Do Not

The published clinical literature supports a clear but limited conclusion. Multiple small-molecule RIPK1 inhibitors can achieve systemic exposure, CNS penetration, and measurable pharmacodynamic target engagement in humans [50,51,60]. These studies represent an important advance from the early use of Nec-1 derivatives because they demonstrate that selective RIPK1 kinase inhibition is technically feasible at exposures relevant to the CNS.

However, the existing published studies primarily address pharmacology and short-term safety rather than therapeutic efficacy. Reduction of pS166-RIPK1 in peripheral blood mononuclear cells demonstrates that a compound engages RIPK1, while measurement of drug in CSF supports CNS exposure. Neither observation establishes that RIPK1 activity has been inhibited in the disease-relevant brain cells, that downstream inflammatory or cell-death programs have been altered, or that such changes will slow neurodegeneration. Accordingly, CNS drug exposure, peripheral target engagement, cerebral target engagement, modification of disease-relevant pathways, and clinical efficacy should be considered distinct and sequential levels of therapeutic evidence.

This distinction is particularly important in AD. The therapeutic hypothesis requires a mechanistic sequence extending beyond drug exposure: the selected patients should have biologically relevant RIPK1 pathway activation; treatment should engage RIPK1 within the appropriate CNS compartment; pathway inhibition should modify inflammatory, lysosomal, vascular, or regulated cell-death processes implicated in AD; and these biological effects should ultimately translate into reduced neurodegeneration or slower clinical progression. Published clinical studies have established the first steps in this sequence but not the latter ones.

### 6.4. Which RIPK1 Function Should Be Targeted: Scaffold, Kinase, or Both?

Most clinically developed RIPK1 inhibitors target kinase activity. This strategy is rational because RIPK1 kinase activity is required for RIPK1-dependent apoptosis and for RIPK1–RIPK3–MLKL necroptosis. Kinase inhibition therefore directly addresses the cell-death arm of RIPK1 biology and may also suppress inflammatory outputs that depend on kinase-active RIPK1 under conditions of checkpoint failure.

However, the AD literature raises a more complex issue. Some disease-associated functions of RIPK1 may not represent classical necroptosis. Ofengeim et al. showed that RIPK1 promotes a disease-associated microglial transcriptional response and lysosomal dysfunction in AD models [15]. Therapeutic benefit in AD could therefore require suppression of microglial inflammatory reprogramming as much as, or perhaps more than, inhibition of terminal necroptosis. An important unresolved question is whether selective kinase inhibition is sufficient to reverse these microglial programs or whether some disease-relevant effects depend on RIPK1 scaffolding, complex formation, ubiquitin regulation, or interactions with additional inflammatory pathways.

A useful therapeutic framework is therefore to distinguish at least two RIPK1 outputs: inflammatory-glial reprogramming and kinase-dependent regulated cell death. These outputs are mechanistically related but not identical. In some AD contexts, RIPK1 inhibition may act primarily by reducing maladaptive microglial signaling and improving Aβ handling. In others, it may protect neurons or endothelial cells from regulated death. At advanced disease stages, both mechanisms may remain active yet no longer be sufficient to restore circuits already lost. This distinction should guide preclinical study design, biomarker selection, and clinical endpoints.

### 6.5. Timing and Patient Selection

The therapeutic success of RIPK1 inhibition in AD will likely depend on disease stage and biological patient selection. If RIPK1 primarily amplifies glial inflammatory dysfunction and lowers the threshold for neuronal injury, inhibition may be most effective before extensive irreversible neuronal loss has occurred. This would favor intervention during prodromal or early symptomatic disease, when Aβ and tau pathology are established but inflammatory and death-signaling pathways may remain modifiable. At later stages, RIPK1 inhibition could suppress active inflammation or cell-death signaling without restoring neuronal networks that have already been lost.

Patient selection is equally important. AD is biologically heterogeneous. Some patients may show substantial Aβ and tau pathology with relatively limited inflammatory or necroptotic pathway activity, whereas others may exhibit prominent microglial, vascular, metabolic, or regulated cell-death signatures. RIPK1 inhibition may therefore be most rational in patients in whom pathway activation can be demonstrated rather than in unselected AD populations.

Combination with Aβ- or tau-directed therapy may also influence timing and patient selection. Anti-Aβ therapies can slow clinical decline in early AD, but their effects are incomplete [61,62]. This creates a conceptual rationale for targeting downstream disease amplification. If Aβ and tau initiate or sustain upstream pathology while RIPK1 contributes to inflammatory injury and regulated cell death, RIPK1 inhibition may ultimately be more useful as an adjunctive strategy than as a replacement for pathology-directed treatment.

### 6.6. Biomarkers of Target Engagement and Pathway Activity

Biomarker development is likely to be one of the most important requirements for RIPK1-targeted therapy in AD. Published clinical studies have used suppression of pS166-RIPK1 in peripheral blood mononuclear cells as a pharmacodynamic measure of target engagement [50,51,60]. This confirms drug–target interaction but does not demonstrate that RIPK1 activity has been suppressed in disease-relevant CNS cells or that AD-relevant inflammatory and death pathways have been modified.

Future AD trials will therefore require biomarkers at several levels. Exposure and target-engagement measures could include CSF drug concentrations together with evidence of RIPK1 kinase inhibition. Pathway-activity biomarkers should ideally capture inflammatory glial states or necroptosis-related signaling within the CNS. Candidate approaches include CSF or plasma inflammatory markers; soluble TREM2 as a microglial-associated marker; extracellular-vesicle measurements of phosphorylated RIPK1, RIPK3, or MLKL; and transcriptomic or proteomic signatures reflecting RIPK1 pathway activity [50,63]. Downstream disease markers should include measures such as neurofilament light chain, tau biomarkers, structural or functional imaging, and cognitive or functional outcomes [64].

Imaging may provide complementary information. TSPO PET and emerging microglial imaging approaches can assess aspects of neuroinflammation in vivo, and TSPO PET signals have been associated with AD pathology and disease-progression models [65]. However, TSPO PET does not directly measure RIPK1 activity and cannot reliably distinguish protective from maladaptive microglial states. Imaging should therefore be incorporated into multimodal biomarker strategies rather than interpreted as a stand-alone measure of RIPK1 pathway activity.

At present, the published literature does not provide a validated in vivo biomarker that directly reports RIPK1 activation in the AD brain. This limitation has major implications for clinical development. Without a CNS pathway-specific biomarker, an unsuccessful therapeutic study could reflect absence of pathway activation in the enrolled population, inadequate engagement in relevant CNS cells, inappropriate disease stage, unsuitable endpoints, or a genuinely ineffective therapeutic mechanism. Biomarker development should therefore proceed in parallel with therapeutic development.

### 6.7. Safety Considerations

Long-term safety represents a major translational requirement for RIPK1 inhibition in AD. RIPK1 participates in host defense, inflammatory responses, cell survival, apoptosis, and necroptosis. Although selective kinase inhibition may preserve important scaffold-dependent functions, chronic inhibition in older adults still requires careful evaluation. Patients with AD commonly have age-related immune dysfunction, vascular and metabolic comorbidities, renal or hepatic impairment, and polypharmacy, all of which can narrow the therapeutic window.

The SAR443060 experience illustrates the importance of distinguishing short-term clinical tolerability from the safety requirements of chronic treatment. Although SAR443060 was generally well tolerated and achieved pharmacodynamic target engagement during early clinical studies, its development was discontinued because of long-term nonclinical toxicology findings [50]. Importantly, those dose-limiting toxicities had not been reported for other RIPK1 inhibitors, leading the investigators to conclude that they were more likely compound-specific than a consequence of RIPK1 inhibition itself [50].

Published studies of SAR443820 and SIR9900 provide reassuring short-term safety data [51,60], but their limited duration cannot establish the safety of prolonged RIPK1 inhibition. This distinction is especially important for AD, in which a disease-modifying intervention could require treatment for years. Future development will therefore require long-duration toxicology, careful assessment of dose–exposure relationships, and clinical monitoring appropriate for chronic modulation of a signaling protein involved in inflammation, cell survival, and host defense.

### 6.8. Combination Strategies

RIPK1 inhibition may ultimately be most useful as part of combination therapy. AD pathogenesis includes Aβ deposition, tau aggregation, synaptic dysfunction, glial activation, vascular injury, metabolic stress, and neuronal loss. A RIPK1 inhibitor would not be expected to remove established Aβ plaques or tau aggregates directly. Its potential value may instead lie in reducing downstream inflammatory amplification and regulated cell-death signaling.

Several combinations are mechanistically plausible. Anti-Aβ therapy combined with RIPK1 inhibition could test whether reducing upstream Aβ burden while suppressing downstream inflammatory and necroptotic amplification produces additive benefit. Anti-tau therapy plus RIPK1 inhibition could be relevant if tau-associated neuronal stress lowers the threshold for RIPK1–RIPK3–MLKL signaling. Microglia-directed therapies could potentially be combined with RIPK1 inhibition if complementary approaches can restore beneficial phagocytic or lysosomal functions while limiting maladaptive inflammatory signaling. Vascular or metabolic interventions may also warrant consideration in biologically defined patients with prominent neurovascular or metabolic dysfunction.

These strategies should be driven by mechanistic evidence rather than therapeutic breadth alone. If RIPK1 functions primarily as a context-dependent amplifier downstream of established Aβ or tau pathology, combining RIPK1 inhibition with upstream pathology-modifying treatment may be more rational than testing it as monotherapy in biologically unselected patients.

### 6.9. Translational Perspective

RIPK1 remains a biologically plausible but clinically unvalidated therapeutic target in AD. Human neuropathology and experimental studies support pathway activation and provide mechanistic evidence linking RIPK1 to microglial dysfunction, inflammatory amplification, neuronal vulnerability, and regulated cell death. Published early-phase clinical studies further demonstrate that brain-penetrant RIPK1 inhibitors can achieve CNS exposure and substantial pharmacodynamic target engagement [50,51,60].

These achievements should nevertheless be interpreted within their evidentiary limits. CNS penetration does not establish target engagement within disease-relevant brain cells, peripheral pS166-RIPK1 suppression does not demonstrate modification of AD biology, and short-term tolerability does not establish the safety of chronic treatment. Most importantly, the published clinical literature does not yet demonstrate that RIPK1 inhibition modifies the progression of AD.

The next phase of RIPK1 development in AD should therefore be hypothesis-driven. Future studies should establish that RIPK1 is active in the targeted disease stage and patient subgroup, demonstrate pathway engagement within relevant CNS compartments, link that engagement to modification of AD-relevant inflammatory or cell-death biology, and establish a safety margin compatible with chronic administration. Only through this sequence will it be possible to determine whether RIPK1 is a therapeutically actionable amplifier of AD pathogenesis or primarily a biomarker of inflammatory and neurodegenerative stress.

## 7. Conclusions

RIPK1 has emerged as a compelling but still incompletely understood signaling node in AD. Its appeal lies in its biological position: RIPK1 connects receptor-proximal inflammatory signaling with downstream decisions that determine whether cells survive, undergo apoptosis, activate necroptosis, or reshape the inflammatory environment. This dual capacity allows RIPK1 to link several processes that are central to AD pathogenesis, including Aβ-associated glial activation, tau-related neuronal vulnerability, chronic cytokine signaling, neurovascular injury, and regulated necrotic cell death.

The central message of this review is that RIPK1 should not be viewed only as a necroptosis marker. In AD, RIPK1 is better understood as a context-dependent signaling checkpoint whose output depends on cell type, disease stage, ubiquitination state, kinase activation, caspase-8 activity, and the surrounding inflammatory milieu. In microglia and astrocytes, RIPK1 may primarily influence inflammatory and disease-associated cellular states; in neurons and vascular cells, it may contribute more directly to regulated death and tissue injury. These functions are not mutually exclusive. Instead, they may cooperate to create self-reinforcing cycles of inflammation, proteostatic stress, cell death, and damage-signal release.

At the same time, the field should avoid overstating the current evidence. Human studies support activation of RIPK1-related pathways in AD, and experimental models support functional roles for RIPK1 in microglial dysfunction, Aβ accumulation, neuronal vulnerability, and necroptosis. However, RIPK1 has not yet been proven to be a primary causal driver of human AD, nor has RIPK1 inhibition been established as a disease-modifying therapy. The most defensible interpretation is that RIPK1 may act as an understudied amplifier and convergence pathway within the broader AD network.

This interpretation provides a rationale for further therapeutic evaluation of RIPK1. If RIPK1 integrates Aβ, tau, inflammation, necroptosis, and vascular stress, then targeting RIPK1 could complement Aβ- or tau-directed approaches by reducing downstream inflammatory and cytotoxic consequences. However, this possibility will require cell-type-specific mechanistic studies, validated biomarkers of pathway activation, rational patient selection, and careful safety monitoring. Establishing whether RIPK1 is a driver, amplifier, biomarker, or therapeutic vulnerability will determine whether RIPK1 inhibition becomes a rational strategy for biologically defined subsets of AD rather than a generic anti-inflammatory intervention.

## Figures and Tables

**Table 1:** Evidence linking RIPK1/necroptosis signaling to Alzheimer’s disease pathogenesis.

Evidence Domain and Model/System	Key RIPK1/Necroptosis Findings and AD-Relevant Implications	Interpretation and Caveats	Evidence Assessment	Reference
Human AD + preclinical inhibition Postmortem AD brain; APP/PS1 and 5 × FAD mice; primary cortical neurons	RIPK1 and MLKL were increased in AD brain, necroptosis markers correlated with disease severity, and pharmacological inhibition reduced neuronal loss and cognitive deficits in AD mice. Supports necroptosis as a candidate mechanism for neuronal loss and memory impairment in AD.	Combines human neuropathological evidence with mechanistic intervention in AD mouse models. Human findings remain largely correlative, and pharmacological results should be interpreted in light of inhibitor specificity and dosing.	Evidence class: Mixed: direct RIPK1 evidence in experimental models; pathway-level evidence in human tissue Study type: Human postmortem + AD mouse + primary neuronal models Causal strength: Moderate: functional pharmacological intervention in models; human evidence remains associative Intervention specificity: Moderate: pharmacological RIPK1/necroptosis inhibition, with inhibitor-specificity caveats Target engagement: Limited/not directly established	[16]
Microglial inflammatory state AD mouse models with pharmacological and genetic RIPK1 inhibition; microglial transcriptomic analyses	RIPK1 promoted a disease-associated microglial program, inflammatory cytokine expression, lysosomal dysfunction, Aβ burden, and memory impairment; RIPK1 inhibition reduced these outcomes. Positions RIPK1 as a regulator of glial dysfunction and Aβ-related inflammation, not only as a necroptosis kinase.	Supports a role for RIPK1 in disease-associated microglial reprogramming and lysosomal dysfunction that is mechanistically distinct from classical RIPK1–RIPK3–MLKL necroptosis.	Evidence class: Direct RIPK1 evidence Study type: AD mouse models + microglial transcriptomics Causal strength: High in experimental models Intervention specificity: High: genetic and pharmacological RIPK1 manipulation Target engagement: Strong functional target validation through RIPK1 manipulation	[15]
Human neuropathology: GVD-necroptosis Postmortem AD, pathologically defined preclinical AD, and non-AD controls	Activated necrosome components pRIPK1, pRIPK3, and pMLKL localized to granulovacuolar degeneration bodies and associated with neuronal loss in vulnerable AD regions. Suggests that necrosome activation may have a recognizable neuropathological substrate in AD neurons.	Provides anatomically resolved human evidence for necrosome activation in vulnerable AD neurons, although postmortem localization remains correlative and does not establish a causal role in neuronal loss.	Evidence class: Pathway-level/indirect evidence Study type: Human postmortem neuropathology Causal strength: Low: associative Intervention specificity: Not applicable: no RIPK1-directed intervention Target engagement: Not applicable; phospho-proteins indicate pathway activation, not intervention target engagement	[40]
TNF/TNFR1-linked neuronal necroptosis Postmortem AD hippocampus; human iPSC-derived glutamatergic neurons	TNF/TNFR1 necroptosis pathway proteins were increased in AD hippocampus, with pRIPK3 and pMLKL mainly in CA1 neurons; inhibition of RIPK1, RIPK3, or MLKL reduced TNF-induced neuronal death in vitro. Links inflammatory TNF signaling to neuronal necroptosis and neuron loss in AD hippocampus.	Provides a mechanistic link between TNF/TNFR1 signaling and neuronal necroptosis; however, necroptosis in vitro required a sensitized cellular context, limiting direct extrapolation to the intact AD brain.	Evidence class: Mixed: direct RIPK1 evidence in vitro; pathway-level evidence in human tissue Study type: Human postmortem + human iPSC-derived neurons Causal strength: Moderate: functional in vitro intervention plus associative human evidence Intervention specificity: Moderate–high: pharmacological inhibition at RIPK1, RIPK3, and MLKL Target engagement: Functional pathway inhibition demonstrated; direct molecular target engagement not established	[41]
Autophagy–necroptosis crosstalk Cell and mouse AD models; TNF-α signaling; RIPK1-p62-UVRAG axis	TNF-α-dependent neuronal necroptosis was regulated by coordination of RIPK1–RIPK3 activity with p62 and autophagic UVRAG; UVRAG overexpression reduced necroptosis in AD models. Connects RIPK1 signaling to autophagic stress, a major pathogenic process in AD.	Supports mechanistic crosstalk between autophagy and RIPK1-dependent necroptosis, although the contribution of the RIPK1–p62–UVRAG axis to human AD remains to be established.	Evidence class: Pathway-level/indirect evidence Study type: Cellular + mouse AD models Causal strength: Moderate for autophagy–necroptosis crosstalk Intervention specificity: Low–moderate for RIPK1: UVRAG was directly manipulated, while RIPK1 was implicated within the pathway Target engagement: RIPK1-specific target engagement not directly established	[42]
Metabolism/O-GlcNAcylation and necroptosis Human AD brain and AD mouse models; biochemical and intervention approaches to modulate O-GlcNAcylation	O-GlcNAcylation was reduced while necroptosis markers were increased in AD; increasing O-GlcNAcylation ameliorated AD-like pathology by suppressing necroptosis. Links metabolic dysfunction, tau/Aβ pathology, and RIPK1–RIPK3–MLKL pathway activation.	Supports an intersection between metabolic/post-translational dysregulation and necroptosis, although the causal hierarchy linking reduced O-GlcNAcylation, AD pathology, and RIPK1–RIPK3–MLKL activation remains unresolved.	Evidence class: Pathway-level/indirect evidence Study type: Human postmortem + AD mouse models Causal strength: Moderate for O-GlcNAcylation–necroptosis coupling; low for RIPK1-specific causality Intervention specificity: Low for RIPK1: intervention targeted O-GlcNAcylation rather than RIPK1 Target engagement: RIPK1-specific target engagement not established	[43]
Aβ oligomers, microglia, and non-cell-autonomous neuronal death Postmortem human AD brain; Aβ oligomer mouse models; Aβo-stimulated microglia–neuron co-culture/conditioned medium systems	Aβ oligomers associated with necroptosis markers in human AD brain. Aβo-stimulated microglia induced neuronal necroptosis through TNF-α signaling; pharmacological or genetic necroptosis inhibition reduced neurodegeneration and memory impairment. Provides a mechanistic link from Aβ to microglial activation to neuronal necroptosis.	Supports a non-cell-autonomous amplification mechanism in which Aβ oligomers activate microglia, leading to TNF-dependent neuronal necroptosis, rather than a model in which Aβ directly activates RIPK1-dependent death in all neurons.	Evidence class: Pathway-level/indirect evidence Study type: Human postmortem + Aβ mouse models + co-culture/conditioned-medium systems Causal strength: High for microglia/TNF-dependent necroptosis in models; RIPK1-specific causality is not isolated Intervention specificity: Moderate: pharmacological/genetic necroptosis-pathway inhibition Target engagement: Pathway-level functional validation; RIPK1-specific target engagement not isolated	[44]
Tau-driven necroptosis and inflammatory signaling Neuronal cell models expressing hyperphosphorylated tau; TauP301S mice; pharmacological and CRISPR approaches	Hyperphosphorylated tau promoted RIPK1–RIPK3–MLKL necrosome formation, NF-κB-dependent cytokine expression, and neuronal death; RIPK1/necroptosis inhibition improved behavioral and inflammatory readouts in tauopathy mice. Places RIPK1 signaling downstream of tau pathology and links neuronal death to cell-autonomous inflammatory programs.	Provides strong mechanistic evidence linking tau pathology to RIPK1–RIPK3–MLKL signaling, although findings from experimental tauopathy models may not fully generalize to sporadic AD.	Evidence class: Direct RIPK1 evidence in experimental models Study type: Neuronal models + TauP301S mice Causal strength: High in experimental tauopathy models Intervention specificity: Moderate–high: pharmacological and CRISPR manipulation of RIPK1/necroptosis signaling Target engagement: Functional pathway suppression supported; scaffold-versus-kinase contribution remains unresolved	[45]
Comorbid TDP-43/LATE-NC and AD neuronal loss Postmortem AD cases stratified by limbic-predominant age-related TDP-43 encephalopathy neuropathological change	Comorbid LATE-NC increased CA1 neuronal loss and aggravated GVD-mediated necroptosis pathway activation in AD. Suggests that RIPK1/necroptosis may integrate AD pathology with common age-related comorbid proteinopathies.	Supports necroptosis as a potential convergence mechanism through which comorbid LATE-NC may exacerbate neuronal loss in AD, but does not establish RIPK1-specific causality.	Evidence class: Pathway-level/indirect evidence Study type: Human postmortem neuropathology Causal strength: Low: associative Intervention specificity: Not applicable: no RIPK1-directed intervention Target engagement: Not applicable	[46]
Vascular/BBB involvement Cerebral endothelial cells; Nat1-deficient mice; two AD mouse models; human hNAT2-related analyses	Reduced mNAT1/hNAT2 promoted endothelial necroptosis, BBB damage, and Aβ accumulation; restoring endothelial mNat1 improved cognitive dysfunction in AD mice. Extends necroptosis/RIPK1-related biology to the neurovascular unit and Aβ clearance.	Extends necroptosis-related mechanisms to endothelial dysfunction and impaired Aβ clearance; however, the extent to which these effects reflect RIPK1-dependent signaling rather than NAT1/hNAT2-specific mechanisms remains to be defined.	Evidence class: Pathway-level/indirect evidence Study type: Endothelial cell + genetic mouse + AD mouse + human analyses Causal strength: High for NAT1-dependent endothelial necroptosis/BBB effects; low for RIPK1-specific causality Intervention specificity: Low for RIPK1: the principal manipulation targeted NAT1 Target engagement: RIPK1-specific target engagement not established	[47]
Human-neuron-specific vulnerability Human or mouse neurons xenografted into an AD mouse brain environment; human AD-related validation; pharmacological and genetic manipulation	Human neurons, but not mouse neurons, developed AD-like tau-related degeneration and neuronal loss. The long noncoding RNA MEG3 was upregulated, induced necroptosis, and neuronal loss was rescued by pharmacological or genetic inhibition of RIPK1, RIPK3, or MLKL. Provides a human-specific mechanism linking AD environment, MEG3, and RIPK1–RIPK3–MLKL-dependent neuronal death.	Provides strong mechanistic evidence for human-neuron-specific vulnerability to RIPK1–RIPK3–MLKL-dependent death, although the xenograft paradigm may not fully reproduce the cellular and temporal complexity of human AD.	Evidence class: Direct RIPK1 evidence Study type: Human-neuron xenograft + pharmacological/genetic pathway manipulation Causal strength: High Intervention specificity: High: direct pharmacological/genetic manipulation of RIPK1, RIPK3, and MLKL Target engagement: Strong functional validation of RIPK1–RIPK3–MLKL dependence	[48]
GVD-necroptosis as targetable neuronal death Tau pathology mouse models; APP-Tau primary neurons treated with AD-derived pTau seeds; necroptosis inhibitors	Activated necroptosis-related proteins appeared in tau pathology models and pTau-seeded primary neurons; necroptosis inhibitors reduced GVD-positive neurons and neuronal loss. Strengthens the idea that a delayed, AD-associated form of necroptosis may be therapeutically targetable.	Provides translational-preclinical evidence that GVD-associated necroptosis is pharmacologically modifiable in tau-related models; however, the relevance and therapeutic tractability of this process in human AD require further validation.	Evidence class: Pathway-level/indirect evidence Study type: Tau mouse models + pTau-seeded primary neurons Study type: Tau mouse models + pTau-seeded primary neurons Causal strength: Moderate–high for necroptosis-associated neuronal loss Intervention specificity: Moderate: necroptosis inhibitors were used, but RIPK1-specific contribution was not isolated Target engagement: Functional pathway modulation; RIPK1-specific molecular target engagement not established	[49]
Clinical translation: SAR443060/DNL747 Phase I/Ib randomized, placebo-controlled studies in healthy volunteers and patients with AD or ALS	SAR443060 was an orally bioavailable, CNS-penetrant RIPK1 inhibitor that reached CSF and showed peripheral target engagement measured by reduced pS166-RIPK1; treatment up to 28 days was generally well tolerated in early studies. Shows that CNS-penetrant RIPK1 inhibition and target-engagement measurement are feasible in humans.	Establishes the feasibility of CNS exposure and pharmacodynamic RIPK1 target engagement in humans, but provides no evidence of clinical efficacy in AD; subsequent discontinuation because of nonclinical toxicology findings limits its interpretation to proof of pharmacological feasibility.	Evidence Class: Direct RIPK1 pharmacological evidence; no evidence of AD clinical efficacy Study type: Phase I/Ib human clinical pharmacology Causal strength: High for pharmacological feasibility; none for AD disease modification Intervention specificity: High: direct RIPK1 inhibitor Target engagement: Demonstrated peripherally by pS166-RIPK1 suppression; CSF exposure shown, but brain-cell target engagement not established	[50]
Clinical translation: SAR443820/DNL788 First-in-human phase I study in healthy adults; CSF sampling and peripheral pharmacodynamic target engagement	SAR443820 is a selective, orally bioavailable, brain-penetrant reversible RIPK1 inhibitor; high inhibition of activated RIPK1/pS166-RIPK1 and CSF exposure supported further CNS clinical development. Represents the newer CNS-penetrant RIPK1 inhibitor program after SAR443060/DNL747.	Demonstrates CNS exposure and pharmacodynamic target engagement with a brain-penetrant RIPK1 inhibitor, but these findings represent pharmacological feasibility rather than evidence that RIPK1 inhibition modifies AD progression.	Evidence class: Direct RIPK1 pharmacological evidence; no evidence of AD disease modification Study type: Phase I human clinical pharmacology in healthy adults Causal strength: High for pharmacological feasibility; none for AD disease modification Intervention specificity: High: selective RIPK1 inhibitor Target engagement: Demonstrated peripherally with pS166-RIPK1 inhibition and supported by CSF exposure; brain-cell target engagement not established	[51]

The evidence assessment reported in the fourth column is qualitative rather than a formal systematic-review risk-of-bias score. Causal strength reflects the extent to which the study design and intervention support causal inference for the stated RIPK1/necroptosis mechanism; intervention specificity reflects how directly RIPK1 or the necroptosis pathway was manipulated; target engagement reflects direct evidence that the intended molecular target/pathway was affected. Abbreviations: AD, Alzheimer’s disease; Aβ, amyloid-beta; Aβo, Aβ oligomers; BBB, blood–brain barrier; GVD, granulovacuolar degeneration; iPSC, induced pluripotent stem cell; LATE-NC, limbic-predominant age-related TDP-43 encephalopathy neuropathological change; MLKL, mixed lineage kinase domain-like protein; pTau, hyperphosphorylated tau; RIPK1/3, receptor-interacting protein kinase 1/3; TNF, tumor necrosis factor; TNFR1, tumor necrosis factor receptor 1.

## Data Availability

No new data were created or analyzed in this study. Data sharing is not applicable to this article.
